# Asthma care based on Chronic Care Model in an aging Asian community

**DOI:** 10.1038/s41533-019-0130-1

**Published:** 2019-05-03

**Authors:** L. F. Zheng, Y. L. E. Koh, U. Sankari, N. C. Tan

**Affiliations:** 1 SingHealth Polyclinics, Singapore, Singapore; 2 SingHealth-Duke NUS Family Medicine Academic Clinical Programme, Singapore, Singapore

**Keywords:** Epidemiology, Asthma

## Abstract

To cope with the higher prevalence of asthma and other non-communicable diseases without compromising on quality of care, a Singapore public primary care institution has adopted the Chronic Care Model (CCM). This retrospective cohort study aimed to describe the proportion of patients with well-controlled asthma (based on Asthma Control Test score ≥20) between 2010 and 2016 in association with their management based on the CCM (which covers the polyclinic clinical information system, self-management measures, system re-design and decision support). Data were retrieved from the Singapore National Asthma Programme (SNAP) and institutional clinical quality databases of eight local polyclinics in eastern and southern Singapore. The data were aggregated, analysed and presented in proportions against monthly polyclinic attendances for asthma. From 2010 to 2016, the total asthma attendances increased by 31% from 27,345 to 35,731, with the highest rise among patients aged ≥60 years. The proportion of patients with good asthma control rose from 71.4% to 80.9%; those who received rescue therapy for acute exacerbations fell from 15.8% to 11.7% and those referred to emergency departments after failed rescue therapy decreased from 0.7% to 0.6%. The proportion of patients with updated asthma action plans increased from 66.7% to 73.4% (proxy for self-management). The overall health and process outcomes of asthma seemed to have improved with multiplex of system-based interventions relating to the introduction of CCM in a public primary healthcare institution in Singapore.

## Introduction

Asthma is prevalent worldwide, affecting 339 million people.^1^ Song et al. reported rising asthma prevalence in three developed Asian nations in Japan, Korea and Hong Kong. They also showed increasing prevalence of older patients with asthma, ranging between 1.3% and 15.3% in Asia, which will continue to rise amidst the aging populations and declining birth rates in many parts of Asia.^2^ Older patients are at increased risk of fatal asthma; the majority of such cases are aged >65 years.^3^ In highly urbanised Singapore at the centre of Southeast Asia, the number of older citizens in its multi-ethnic Asian population is expected to double from 440,000 to 900,000 by 2030, as they rank among the top globally in longevity.^4^ With increasing lifespan of these older people with asthma, the disease burden on its healthcare system is postulated to rise significantly.

**Fig. 1:**
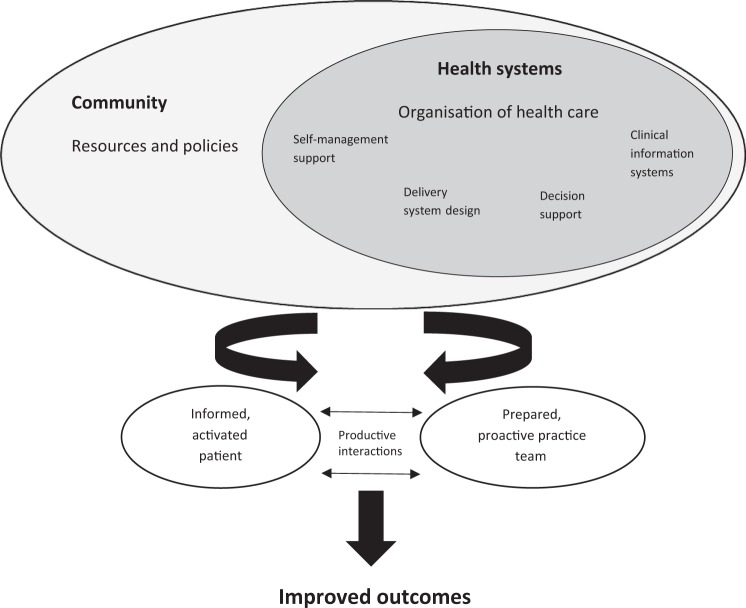
Chronic Care Model developed by The MacColl Institute, © ACP-ASIM Journals and Books, reprinted with permission from ACP-ASIM Journals and Books

Singapore is classified by the World Health Organisation as a first-world nation with a well-established healthcare system.^5^ Nonetheless, asthma mortality in Singapore was triple that of other developed nations in the previous decade.^6^ Increasing age, male gender and Malay ethnicity were identified as risk factors for mortality in its local population.^7^ Acute asthma exacerbations usually precede asthma-related deaths. Local patients can access rescue therapy to relieve their acute exacerbations at any private (GP clinics) or public primary care clinics (polyclinics), which are often located in close proximity to their residences. Those who fail to relieve the exacerbations despite the initial rescue therapy at these clinics will be transferred to the emergency departments in hospitals for further management. Being a major provider of asthma care in the community, optimising acute asthma management in a public primary healthcare institution may potentially mitigate subsequent risks of asthma morbidity and mortality. SingHealth Polyclinics (SHP) is a large cluster of public primary care clinics (polyclinics) spread over the eastern and southern regions of Singapore. Its network of high patient-volume polyclinics served 1.7 million patients with subsidised healthcare services in 2016. Since 2004, the institution has adopted Chronic Care Model (CCM) in the management of non-communicable diseases (NCD), such as asthma. The CCM is a multi-faceted model of care underpinned by clinical information system, self-management support, delivery-system design and decision support,^8^ as shown in Fig. 1. The goal is to leverage on these tenets to develop and enhance the interactions between informed, activated patients with healthcare practice teams who are prepared and proactive. This care model has demonstrated reduced healthcare costs and lower use of healthcare services in the management of NCD.^9^ Its effectiveness has yet to be demonstrated in an Asian population with asthma, especially in a developed nation with significant level of asthma mortality.

The Singapore National Asthma Programme (SNAP) was launched in 2001^10^ to address prevalent fatal asthma. Its extension to primary care in 2002 provided an opportunity and a platform for collection of asthma data to review the quality of care across the public primary to tertiary healthcare institutions. SHP have leveraged on SNAP to complement the polyclinic clinical information system to collect aggregated care quality data of patients with asthma. Amidst the adoption of CCM and in the context of an aging population, this presented an opportunity to assess the magnitude of the asthma burden and quality of asthma care in terms of the demography of asthma attendances and related health outcomes.

Hence, this study aimed to evaluate the outcomes of asthma management in a large public primary care institution in Singapore over 7 years after its adoption of CCM. The outcomes included the proportion of patients with asthma action plans (process outcomes for self-management support), the proportions with well-controlled asthma and those receiving rescue therapy for exacerbations during their consultations (health outcomes) and the number of nurses who underwent specific asthma-related training (surrogate indicator of development of prepared practice teams in each polyclinic).

## Results

The overall asthma attendances in SHP increased by 30.7% from 27,345 in 2010 to 35,731 in 2016, of whom 60.3% were female. The actual number of patients who attended for asthma rose by 26.3% from 11,683 in 2010 to 14,755 in 2016, of whom 55.3% were female. The total patient attendances in SHP also increased by 13.5% from 1,660,044 in 2010 to 1,884,051 visits in 2016 (all are unpublished data from SHP).

**Fig. 2:**
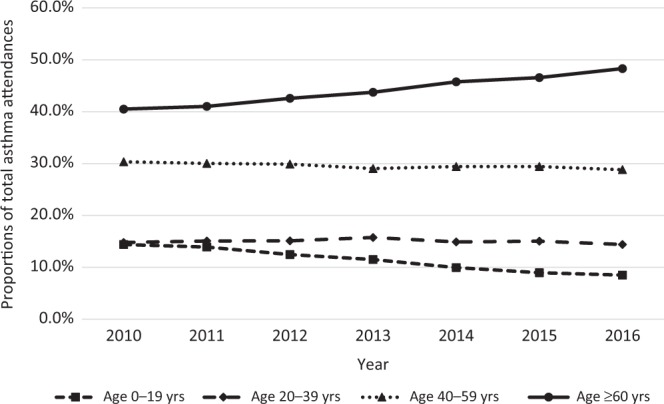
Proportion of asthma attendances by age groups

Among the different age groups, the highest rise was among patients aged ≥60 years, as shown in Fig. 2. Their attendances increased from 40.5% in 2010 to 48.3% in 2016.

The ethnic composition of the attendances were: Chinese (53.2% in 2016), followed by Malay (26.2%), Indian (14.0%), and Others (6.6%) patients. Of the attendances in 2016 for asthma contributed by patients of Malay ethnicity, 40.9% were by male patients. Patients of Malay ethnicity constituted a disproportionately high proportion of asthma attendances though they only made up about 13% of the population in Singapore.

**Fig. 3:**
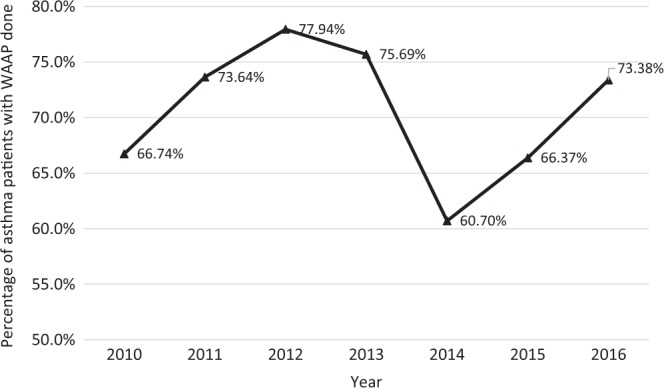
Percentage of asthma patients with Written Asthma Action Plan (WAAP) done in SingHealth Polyclinics

The percentage of patients in SHP who received updated written asthma action plan from their physicians increased from 66.7% in 2010 to 73.4% in 2016, as shown in Fig. 3.

**Fig. 4:**
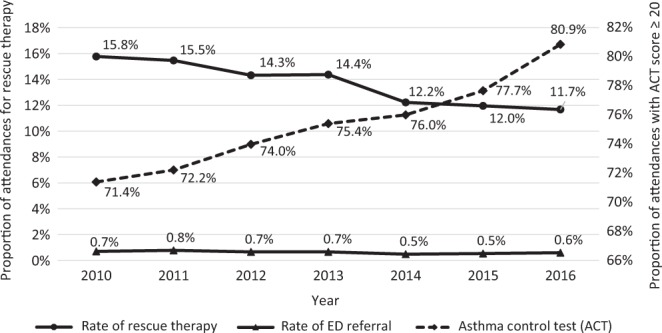
Markers of control as a proportion of asthma attendances

Amid rising attendances, the proportion of patients who achieved good asthma control (Asthma Control Test (ACT) score ≥20) has risen from 71.4% in 2010 to 80.9% in 2016, as shown in Fig. 4.

The proportion of patients receiving rescue therapy for acute asthma exacerbation fell from 15.8% in 2010 to 11.7% in 2016. Similarly, the proportion of patients with asthma referred to emergency departments in secondary care after failed rescue therapy remained stable, ranging from 0.7% in 2010 to 0.6% in 2016.

**Fig. 5:**
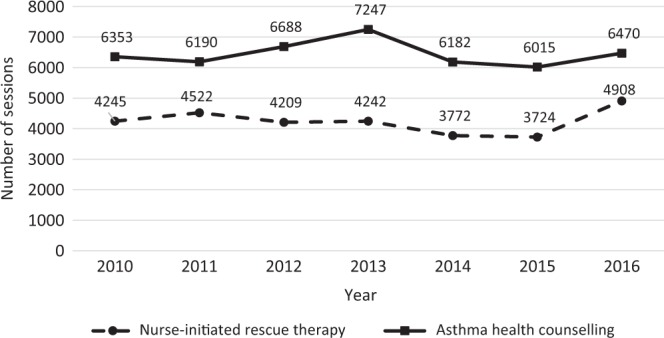
Number of nurse-initiated rescue therapy and asthma counselling

As a proactive practice team, the polyclinic nurses were able to initiate protocol-based rescue therapy based on clinical assessment before patients were seen by doctors. From 2010 to 2016, a total of 72 registered nurses underwent training and certification for rescue therapy initiation and asthma health counselling. As a result, the number of nurse-initiated rescue therapy increased by 15.6% from 4245 cases in 2010 to 4908 cases in 2016; the number of asthma health counselling sessions done by nurses also rose by 1.8% from 6353 sessions in 2010 to 6470 sessions in 2016, as shown in Fig. 5.

## Discussion

The results showed rising attendances for asthma from 2010 to 2016, comprising mainly patients aged ≥60 years. The higher attendances by older patients with asthma could be attributed to their consultations for other co-morbidities, but data were captured owing to concurrent disease coding for the same visit. The extra consultation and medication subsidies for the older patients aged 65 years under the local healthcare finance policy impacted the selection of their care providers in the public polyclinics.^11^

A disproportionately high proportion of patients of the Malay minority ethnic group, as compared to their national ethnicity composition, attended the polyclinics for asthma. A higher smoking prevalence among patients of Malay ethnicity, potentially increasing their risks of poor asthma control, could be one associated factor. The 2010 Singapore National Health Survey found that adult Malays, aged 18–69 years, had the highest daily smoking prevalence (26.5%), compared to Chinese (12.8%) and Indians (10.1%).^12^ However, the database in this study did not provide information on the smoking status in patients with asthma in SHP.

The proportion of the patients who attained ACT scores of ≥20 increased year on year from 2010 to 2016. This corresponded with a decline in rescue therapy and referrals to the emergency department. The adoption of CCM in SHP underpins the general improvement in asthma care in its patients.^13–15^ CCM is the institutional framework to optimise chronic disease management via delivery system design, clinical information system, decision support and self-management support. The goal is to facilitate the development of a prepared and proactive practice team in each of its polyclinics to engage and interact with informed, activated patients.

Nevertheless, this model of care caters to the general patient population, regardless of various subsets of the asthma population with special needs. The co-morbidities of the older patients with asthma and those of Malay ethnicity with higher smoking prevalence will require additional attention to their associated risk factors. Beyond the CCM, the institution has trained and introduced case and care managers with nursing background in the polyclinics to manage patients with special needs and medical complexity in 2016. The aim is to address the remaining 20% of patients who have acute exacerbations and those who have yet to achieve satisfactory asthma control (based on ACT score) during their polyclinic visits. This health service-related intervention is currently being evaluated for its cost-effectiveness.

This report also reflects clinical quality in real-world setting. Figure 3 showed a decline in the proportion of patients receiving updated written asthma action plan from their physicians. A shortage of physicians in the institution in 2014 led to reduced provision of updated asthma action plan to the patients. The indicator increased in subsequent years after the manpower exigency abated.

This study has a number of limitations. The results consist of aggregated data that have been presented as patient attendances, limiting the longitudinal study of their outcomes. Patients with poor asthma control tend to be reviewed more frequently and inevitably generate a higher number of attendances at the polyclinics. Moreover, those who were assessed by the nurse-led telehealth service can obtain their prescriptions without a physician consultation. Thus the aggregated data may potentially underestimate the proportion of patients with satisfactory asthma control.

The SHP SNAP database did not collect prescription data, except for two of its polyclinics due to resource limitations. The two selected polyclinics were the sites for the experiment to determine whether additional subsidy of asthma medications would lead to better outcomes.^16^ The increasing use of controller medications, especially the use of inhaled corticosteroid long-acting beta-agonist medications at these two sites, mirrored the trend for the rest of the polyclinics, which may impact on the asthma control status of the patients, as reflected in their ACT scores^17^ (Fig. 4).

The data are largely based on physician-diagnosed asthma in the electronic medical record (EMR). Spirometry-based asthma diagnosis is limited to few patients in the EMR, when they were previously investigated in secondary or tertiary healthcare institutions. Nonetheless, the polyclinic physicians are guided by local and international asthma clinical practice guidelines and referral for confirmation by pulmonologist by spirometry is integral in their practice. With the introduction of spirometry at SHP from April 2018 using a mobile team of trained respiratory laboratory technicians from a tertiary institution, the accuracy of asthma diagnosis will be enhanced.

Process indicators to capture every aspect of CCM implementation are limited. Going forward, new initiatives and programmes that are introduced in the institution to enhance asthma care will be evaluated using relevant process indicators based on implementation science or quality improvement methodology.

## Methods

### Study sites

The data for this study originated from the eight polyclinics of SHP from 2010 to 2016 before the structuring of the local primary healthcare system in 2017.

### Model of care at the study sites—CCM

All eight sites of SHP adapted CCM to their asthma care processes (Table 1).

**Table 1:** Adaptations of CCM to SingHealth Polyclinics

Key pillars of the Chronic Care Model	Adaptations
System re-design	Health Monitoring Stations (HMS) are set up at each polyclinic, in which trained Allied Healthcare Workers (AHW) use the Asthma Control Test (ACT) to triage patients with asthma. The ACT scores for patients with asthma constitute a key clinical performance indicator for each polyclinic. The indicator is collated monthly by the institution Quality Management department and presented to the director of each polyclinic during senior management meeting as one of the measures for clinical performance
Clinical information system	The ACT scores from the HMS are entered into the electronic medical records (EMRs) for review by the physicians and nurses. The ACT scores are accessible to physicians and nurses at their service points. The data depicts the status of the current visit as well as those of previous visits; they are presented as data points or in the form of trend chart to reflect longitudinal asthma control status
Decision support	Clinicians such as physicians and nurses gain an overview of the asthma control of each patient from the asthma dashboard in the EMR. The dashboard is automated as the first interface when clinicians access the EMRs of patients with diagnosis of asthma. Clinical data such as the ACT score, PEFR measurements, smoking status, and asthma action plan on the dashboard facilitate decision support for the physicians to review the treatment plan
Proactive practice team	Patients identified by AHWs with acute asthma symptoms and/or low ACT scores on arrival at the polyclinic or at the HMS will be immediately directed to the trained nurses at the polyclinics for assessment before patients are further reviewed by physicians. Every registered nurse will be rostered to undertake specific training module, including lung auscultation technique, based on a protocol-based rescue therapy regimen
Proactive practice team	Multidisciplinary team of primary healthcare professionals from all the polyclinics, meets quarterly to review the care processes, shares best practices and makes recommendation to improve care services to patients with asthma. Minutes are taken at these collaborative meetings for dissemination to other staff
Self-management support	Each patient (or caregiver of paediatric patients) is provided with a personalised written asthma action plan, which is updated at each visit. The action plan is a self-management platform for the multidisciplinary team to educate and remind patients of their adherence to their medications according to their asthma control status and to self-initiate rescue therapy at the advent of impending asthma exacerbation. Oral prednisolone is prescribed for adult patients as stand-by medication for use in acute exacerbations

*CCM* Chronic Care Model, *PEFR* peak expiratory flow rate

As part of system re-design, SHP has introduced health monitoring stations operated by allied healthcare workers at each polyclinic to assess and triage patients with asthma using the ACT. The ACT scores are entered into the EMRs, which are part of the clinical information system, and the information is available to physicians during the consultation as a form of decision support. The clinicians (physicians and nurses) gain an overview of the patient’s asthma control and management quickly from an asthma dashboard in the EMR. The dashboard is automated as the first interface when clinicians access the EMR of patients with asthma. An ACT score of ≥20 constitutes good asthma control. Patients detected with acute asthma symptoms will be immediately reviewed by trained nurses at the polyclinics. They will initiate protocol-based rescue therapy promptly before patients are further reviewed by physicians, demonstrating the concept of a proactive practice team. The nurses and pharmacist will support the physicians in educating patients on their personalised written asthma action plans for self-management and assessing their inhaler technique. The clinical information system allows the ACT and asthma action plan to be incorporated into the EMR, from which data of its provision to patients can be generated.

In addition, another example of being a proactive practice team, a multidisciplinary team of primary healthcare professionals meets quarterly to review the care processes and makes recommendation to improve care services to patients with asthma and other respiratory diseases. They are guided by local and GINA clinical practice guidelines.^18^ The institution tracks the proportion of patients achieving satisfactory asthma control based on ACT scores from their EMR. Such quality data are shared across polyclinic senior management every month. Local quality improvement teams are organised at the polyclinics to identify the barriers and test solutions to overcome the gaps.

### Study population

#### Inclusion and exclusion criteria

The study population comprised of patients with a diagnosis code of asthma in their EMR from 1 January 2010 to 31 December 2016. They were included if they had at least one attendance with the diagnosis “asthma” recorded in the clinical information system in any polyclinic within the institution from 1 January 2010 to 31 December 2016.

Patients without diagnosis code of asthma and those who had a concomitant diagnosis of chronic obstructive pulmonary disease were excluded from the study.

#### Data retrieval from SHP SNAP Database

SNAP allows each public healthcare institution to develop its respective database due to the different EMR systems. The SNAP database in SHP was designed to collect aggregated data based on asthma attendances using the diagnosis codes from the EMR system. Every month, the assigned asthma nurse in each polyclinic collated the data on the number of patients who received rescue therapy for acute asthma, the age, gender, race of the patients and the immediate outcome of rescue therapy, such as discharge or referral to emergency department. Subsequently, the asthma nurses sent the data to the SNAP coordinator and the data clerk for compilation each month. Data on the total number of patient attendances, including those for asthma, as well as the ACT scores were retrieved from the institution business operation and EMR systems.

#### Statistical analysis

The total monthly attendances in the 8 polyclinics for any consultation and specifically for asthma constituted the denominators of the data computations. Patient demographic data, their ACT scores, number who received rescue therapy for acute asthma and immediate outcomes of rescue therapy (resolved and discharged or referral to emergency department of nearest hospitals) were collated, analysed and presented as charts in Microsoft Excel.

#### Ethics approval

The study was approved by the SingHealth Centralised Institutional Review Board (CIRB approval number 2016/2099).

## Data Availability

Anonymised data are available on request from the authors.
